# Assessment of Gastric Accommodation in Patients with Functional Dyspepsia by 99mTc-Pertechtenate Single Photon Emission Computed Tomography Imaging: Practical but not Widely Accepted

**DOI:** 10.4274/mirt.36854

**Published:** 2015-11-02

**Authors:** Taghi Amiriani, Hamid Javadi, Tahereh Raiatnavaz, Ali Mahmoud Pashazadeh, Shahriar Semnani, Seyed Masoud Tabib, Majid Assadi

**Affiliations:** 1 Golestan University of Medical Sciences (GUOMS), Golestan Research Center of Gastroenterology and Hepatology, Gorgan, Iran; 2 Bushehr University of Medical Sciences, The Persian Gulf Nuclear Medicine Research Center, Bushehr, Iran

**Keywords:** Gastric accommodation, functional dyspepsia, single photon emission computed tomography, fasting gastric volumes, postprandial gastric volume

## Abstract

**Objective:**

Impaired gastric accommodation is one of the main symptoms in patients with functional dyspepsia. The aim of the present study was to assess gastric accommodation in patients with functional dyspepsia using single photon emission computed tomography (SPECT) imaging.

**Methods:**

Twenty-four patients with functional dyspepsia and 50 healthy volunteers as control group were enrolled in this study. All participants were given 5 mCi 99mTc-pertechtenate intravenously, served with a low fat meal, and underwent SPECT scanning 20 minutes after the meal.

**Results:**

Based on the scintigraphic data, gastric volumes were found to be significantly increased after food ingestion in both patient and control groups. We also found that while there was no significant difference between patient and control groups in terms of fasting gastric volumes, postprandial gastric volume was significantly lower in patients as compared to healthy individuals (p<0.05).

**Conclusion:**

Measuring gastric volume by using SPECT can be a valuable method in the detection of functional dyspepsia and in differentiation of this entity from other organic disorders.

## Abstract (tr)

**Amaç:**

Bozulmuş gastrik akomodasyon fonksiyonel dispepsi hastalarının ana belirtilerinden biridir. Bu çalışmanın amacı, fonksiyonel dispepsi hastalarında gastrik akomodasyonun bilgisayarlı tek foton emisyon tomografisi (SPECT) görüntüleme ile değerlendirilmesidir.

**Yöntem:**

Fonksiyonel dispepsisi olan 24 hasta ve kontrol grubu olarak 50 sağlıklı gönüllü çalışmaya alındı. Tüm katılımcılara intravenöz 5 mCi 99mTc-perteknetat uygulandı, düşük yağlı yemek verildi ve yemekten 20 dakika sonra SPECT taraması yapıldı.

**Bulgular:**

Sintigrafik verilere göre gıda alımından sonra hem hasta hem de kontrol gruplarında mide hacimlerinin önemli ölçüde arttığı saptandı. Açlık mide hacimleri açısından hasta ve kontrol grupları arasında anlamlı bir fark saptanmadı, ancak hastalarda tokluk mide hacmi sağlıklı kişiler ile karşılaştırıldığında anlamlı olarak düşük bulundu (p<0,05).

**Sonuç:**

SPECT kullanılarak mide hacmi ölçümü fonksiyonel dispepsinin saptanmasında ve bu patolojinin diğer organik bozukluklardan ayrımında değerli bir yöntem olabilir.

## INTRODUCTION

Gastric accommodation (GA) is a vagal nerve mediated reflex, which is associated with reduction in gastric tone along with an increase in gastric volume and gastric compliance (1). This reflex is a predictable response in healthy subjects that allows ingestion of food without inducing postprandial symptoms. Impaired GA may be observed in many pathologic conditions such as functional dyspepsia, dyspepsia after fundoplication or gastric surgery (2,3). Therefore, assessment of GA may improve our understanding of the causes of upper gastrointestinal symptoms after food intake in functional and neuropathic diseases, and ultimately their effects on the outcome of treatment.

Since the total volume of the stomach is mainly determined by the volume of its proximal portion, changes in proximal gastric volume after eating is assumed to reflect changes in the whole stomach. Evaluation of proximal gastric motor function is of particular importance in clinical trials, as it can be used to detect abnormalities in the stomach, which in turn may be used as an indicator of upper gastrointestinal problems (4,5,6).

The gold standard for measuring GA is the gastric barostat (7). Currently, this is the only reliable method for the assessment of gastric accommodation. A polyethylene balloon is placed in the proximal stomach to measure fasting and postprandial gastric volume. Although this invasive technique is valuable, it is not practical for research studies and is unacceptable for most patients in daily clinical practice. That is why many attempts have been made to identify the role of noninvasive methods such as abdominal ultrasonography and MRI that could replace this invasive procedure (8).

One of the non-invasive methods used to evaluate GA is single photon emission computed tomography (SPECT) using technetium-99m pertechnetate, which was first developed by Camilleri and colleagues at the Mayo medical center (1). Dynamic scintigraphy of the stomach by 99mTc enables measurement of gastric volume using two-dimensional scintigraphic images, and provides images of gastric mucosa and gastric wall motion (9). Several studies have assessed the validity of this method, indicating that there was no significant difference between scintigraphic data and gastric barostat in terms of GA criteria including fasting gastric volume, postprandial gastric volume and the ratio of these two volumes (6,10,11).

For these reasons, in this study we investigated the correlation of functional dyspepsia and GA using SPECT imaging. Measuring GA and changes in gastric volume in functional dyspepsia may lead to better treatment planning for these patients.

## MATERIALS AND METHODS

### Participants and Study Design

Informed consent was obtained from all patients and the Research Ethics Committee of Golestan University of Medical Sciences reviewed the study. Twenty-four patients diagnosed with functional dyspepsia and 50 healthy volunteers as control group were included in this study. The sample size was calculated based on a previous study (2) with α=0.05, β=0.2 and power=80%. Patient data were recorded in the Research Center of Gastroenterology and Hepatology (GRCGH), and they were referred to the Nuclear Medicine center for measurement of fasting and postprandial gastric volumes. All patients had at least two of the following symptoms for more than three months: abdominal discomfort or pain, early satiety, distension, bloating, nausea, vomiting, belching.

Patients with a history of peptic ulcer disease, scleroderma, biliary abnormality, cerebrovascular accidents, diabetes, gastric outlet obstruction, use of corticosteroids, NSAIDs or other drugs with known complication of peptic ulcer, gastritis or gastrointestinal infection, abdominal surgery, metabolic disease and recent trauma to the abdomen were excluded from the study.

Healthy control participants did not have any history of gastrointestinal surgery or gastrointestinal complaints and were not taking any medications.

All participants, both patients and control subjects, had normal findings on esophageal and gastric endoscopy.

### Imagining Protocols

In order to determine GA using SPECT imaging, each individual was administrated intravenously with 5 mCi 99mTc-pertechtenate and then served with a low fat meal including egg. The scintigraphic imaging was performed at fasting and 20 minutes after ingestion of meal to measure gastric volume.

The images were obtained on a large field-of-view, dual-head gamma camera (Prism 1000XP) with a 15% energy window centered on 140 keV, and a low-energy all-purpose collimator.

The upper and lower limits of the stomach were identified by projection of the longest fundus-to-antrum distance. 3-dimensional renderings of the stomach were produced from the SPECT images, and gastric volume was measured by summation of all gastric voxels for each transaxial slice (11).

### Statistical Analysis

The data are represented as mean ± standard deviation. The t-test, Chi-square test and Fisher’s exact test were used when appropriate to determine statistical differences between groups. p-values less than 0.05 were considered as significant. Statistical analysis was performed using an IBM computer and PASW software, version 18.0 (SPSS, Inc., Chicago, IL).

## RESULTS

In this study, 24 patients with functional dyspepsia were compared with 50 healthy subjects, as control group, in terms of GA based on fasting and postprandial gastric volume as determined by SPECT imaging. Demographic data of the subjects, both controls and patients, are presented in Table 1.

Participating individuals (patients and volunteers) in this study were composed of 15 men (20.3%) and 59 women (79.7%). With respect to age, height and weight they ranged from 21 to 67 years (mean of 47.17±12.64 years), 154 to 181 cm (mean of 163.37±7.41) and 40 to 102 kg (mean of 66.28±12.21 kg), respectively. Their mean body mass index (BMI) was 24.69±3.17 kg/m2. Three individuals (4.1%) were underweight (BMI<18.5), and 36 (48.6%) were within the normal range (18.5≤BMI<25), while 30 (40.5%) were over-weighted (25≤BMI<30) and 5 (6.8%) were obese (BMI≥30).

Table 1 shows comparison of demographic characteristics between the two groups. Based on statistical analysis, there was no significant difference between patients and control group in terms of BMI (p>0.05). However, differences between the two groups in terms of age and sex were statistically significant (p<0.0001).

**Figure 1:**
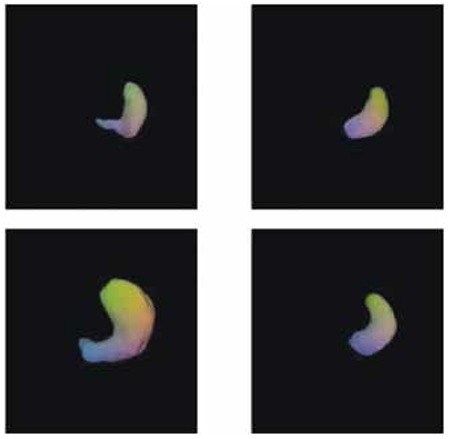
Examples of single photon emission computed tomography images of fasting gastric volumes (top row) and postprandial gastric volumes (botton row) in healthy individuals (left column) and patients with functional dyspepsia (right column)

Results of SPECT imaging to determine fasting and postprandial gastric volumes are given in Table 2. The mean fasting and postprandial gastric volumes determined by GA scintigraphy were 217.54±32.37 and 651.87±70.10 mL in the patient group and were 207.84±13.54 and 760±77.41 mL in the control group, respectively. Statistical analysis of the data showed a significant difference between fasting and postprandial gastric volumes in both patient (p<0.0001) and control group (p<0.0001).We also found that while there was no significant difference between fasting gastric volumes of patients and control group (p>0.05), postprandial gastric volumes were statistically different between these two groups (p<0.0001). Figure 1 presents examples of fasting and postprandial gastric volumes in patients with functional dyspepsia and healthy individuals.

Correlation of fasting and postprandial gastric volumes with demographic properties of patient and control groups were assessed and presented in Table 3. We observed that for patients, both fasting and postprandial gastric volumes were not correlated with age or sex (p>0.05), but correlated with BMI (p<0.05). In the control group, fasting gastric volume was not correlated with age and BMI (p>0.05), but there was a correlation between fasting volume and sex (p<0.05). In addition, postprandial gastric volume of the healthy volunteers were statistically correlated with age, sex and BMI (p<0.05).

## DISCUSSION

The incidence of functional gastrointestinal disorders has been increasing as compared to organic diseases in recent years. In recent years, there has been an increase in the incidence of functional gastrointestinal disorders in comparison with organic diseases. However, studies on this pathology have been limited in clinical practice due to the heterogeneity in the pathogenesis of functional dyspepsia. However, there are still limited number of clinical studies on this pathology because of the heterogeneity in the pathogenesis of functional dyspepsia. Reduced GA is a major pathophysiological mechanism that is usually associated with early satiety, discomfort after eating and weight loss. One of the major pathophysiological mechanisms is the reduced gastric accommodation, that is usually associated with early satiety, discomfort after eating and weight loss. Identification of the correlation between gastric compliance and dyspepsia symptoms is important to understand disease pathogenesis. Ongoing studies aim at improving diagnostic and therapeutic procedures related to this pathology. Several studies have indicated the clinical potential of SPECT imaging in GA evaluation. SPECT generates valuable information on motor abnormalities, providing additional insight into the pathophysiology of functional dyspepsia (1). Scintigraphic data would assist in selective and effective therapy of these patients (1). Vijayvargiya et al. (12) determined that 1 scan after ingestion provided equivalent information on GA as two scans after food intake, and that the difference in method precision was less than 2%. Vasavid et al. (6) reported that measurement of GA by 99mTc-pertechnetate SPECT is associated with favorable reproducibility at both the same and different times in a day. Results of a study performed by Van den Elzen et al. (13), on 21 healthy volunteers that compared results of SPECT with the gold standard barostat, showed that although SPECT scanning was able to detect changes in postprandial gastric volume it was not as suitable as barostat in detecting changes in gastric tone. Simonian et al. (11) showed that SPECT scanning provided an opportunity to measure gastric emptying and gastric accommodation, simultaneously. The present study was in fact an attempt to assess the clinical value of SPECT as a possible alternative to barostat in GA studies.

This study showed that while there was no significant difference in fasting gastric volume between patients and healthy group, the difference between these two groups was statistically significant with respect to postprandial gastric volume. Results of our study on fasting volume were in accordance with the findings by Bredenoord (1), however, our results on postprandial volume contradicted their findings. One possible reason for this controversy may be related to the difference in the number of cases between the two studies.

Published results have indicated that GA is significantly reduced in patients with functional dyspepsia as compared to healthy individuals. This apparent difference was also revealed in our study with findings on postprandial gastric volumes (651.87±70.10 versus 760±77.41). In a study that assessed gastric volume using SPECT on 10 healthy volunteers by Kuiken et al., (14) the mean fasting and postprandial gastric volumes were reported as 182±11 mL and 690±32 mL, respectively. An increase of 200% to 400% was observed in gastric volumes, in both the proximal and distal segments. In our study, the mean fasting and postprandial gastric volumes in healthy subjects were 207.84±13.54 and 760±77.41 mL, respectively, that was higher than corresponding values in their study. Kim et al. (8) reported the postprandial/fasting gastric volume ratio in healthy individuals as 4.9 and stated that 41% of patients with idiopathic non-ulcer dyspepsia showed reduced accommodation, while in our study the postprandial/fasting gastric volume ratio was determined as 3.6 in the control group. A major factor that could have affected the results of our study in comparison to previous studies may be the method that was used to measure gastric volumes. However, the utilization of this noninvasive, clinically applicable scintigraphic test that could simultaneously measure both gastric emptying and accommodation may be valuable in defining the pathophysiology of symptoms in patients with functional dyspepsia, who constitute main referrals to GI departments (11,15).

## CONCLUSION

This study showed that there is a statistical correlation between reduction in gastric volume and postprandial GA with functional dyspepsia symptoms. Furthermore, the adaptive response of the gastric volume, especially postprandial gastric volume, was correlated with age, sex and BMI that was significant in comparison to fasting gastric volume. Therefore, it can be concluded that measuring gastric volume can be a valuable method in the detection of functional dyspepsia and differentiation of this entity from other organic disorders, and that SPECT can be accepted as a feasible method to measure gastric volume changes in response to food intake.

## Figures and Tables

**Table 1:**
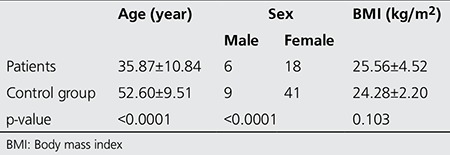
Statistical difference in age, sex and body mass index of patients and control group

**Table 2:**
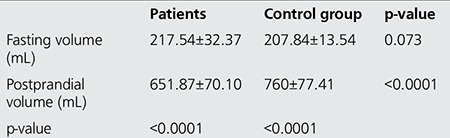
Gastric accommodation in terms of fasting gastric volume and postprandial gastric volume in patients and control group

**Table 3:**
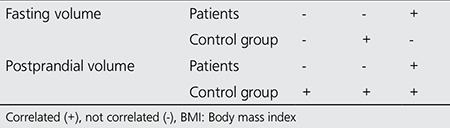
Correlation of fasting and postprandial gastric volumes with demographics of patients and control group
